# Virus-mediated gene transfer of soluble amyloid precursor protein-alpha via systemic injection in a mouse model of Alzheimer’s disease

**DOI:** 10.1038/s41434-026-00602-8

**Published:** 2026-03-03

**Authors:** Yuanyuan He, Bruce G. Mockett, Lucia Schoderboeck, Kirstin O. McDonald, Barbara J. Logan, Shruthi Sateesh, Owen D. Jones, Stephanie M. Hughes, Wickliffe C. Abraham

**Affiliations:** 1 Department of Physiology, University of Otago, Dunedin, New Zealand; 2 Brain Health Research Centre, University of Otago, Dunedin, New Zealand; 3 Department of Psychology, University of Otago, Dunedin, New Zealand; 4 Department of Biochemistry, University of Otago, Dunedin, New Zealand

**Keywords:** Neurotrophic factors, Neurological disorders, Gene delivery

## Abstract

Alzheimer’s disease (AD) is the most common neurodegenerative disorder, yet effective preventive or therapeutic strategies remain limited. A hallmark of AD pathology is the accumulation of insoluble amyloid-β (Aβ) aggregates, which are targeted by recent antibody-based therapies. Conversely, soluble amyloid precursor protein-alpha (sAPPα), a non-amyloidogenic cleavage product of APP, possesses neuroprotective, neurotrophic, and synaptogenic properties, and the ability to enhance memory. This study evaluated the therapeutic efficacy of adeno-associated virus variant PHP.eB (AAV-PHP.eB) encoding human sAPPα in the APPswe/PS1dE9 transgenic mouse model of AD. Six-month-old female wild-type and transgenic mice received a single intravenous injection via the tail vein. Three months post-injection, brain tissue was harvested for electrophysiological and histological analyses. The treatment significantly increased cortical sAPPα levels and fully restored hippocampal long-term potentiation (LTP) in transgenic mice. Post-mortem analyses revealed a substantial reduction in amyloid plaque burden in both the hippocampus and cortex, with minimal plaque progression from the time of injection. However, no significant changes were observed in astrocytic (GFAP) or microglial (Iba-1) coverage, nor in soluble and insoluble Aβ1-40 or Aβ1-42 levels. These findings suggest that systemic AAV-PHP.eB-mediated sAPPα delivery can ameliorate synaptic dysfunction and aggregated amyloid pathology in AD, highlighting its potential as a therapeutic strategy.

## Introduction

Alzheimer’s disease (AD) is the most prevalent neurodegenerative disorder and exhibits a well-defined pattern of neuropathology and behavioural impairments that correlate with progressive loss of brain functionality. Rising human life expectancy has led to a growing incidence of AD. Thus, effective therapeutic approaches are urgently required. However, most of the available pharmacological treatments only alleviate some of the symptoms of AD with modest benefits [1]. According to the amyloid cascade hypothesis, the primary trigger of synaptic dysfunction, neuroinflammation, neuronal degeneration, and the onset of tau pathology is the extracellular accumulation of amyloid-β (Aβ) aggregates. Therefore, re-establishing a balance between Aβ synthesis and clearance remains a promising approach to address the treatment of AD [2, 3]. For this reason, more recent therapies have centred on anti-amyloid antibodies for AD. The anti-amyloid antibodies donanemab and lecanemab have shown promising benefits in clinical trials, although their long-term benefits and safety profiles remain to be fully understood [4].

Employing a different strategy, we have focused on the α-secretase-mediated cleavage product of amyloid precursor protein (APP), namely the N-terminal fragment soluble amyloid precursor protein-alpha (sAPPα). Importantly, the α-secretase-mediated cleavage occurs within the Aβ region of APP. sAPPα is recognised as having therapeutic potential due to its strong neuroprotective, neurotrophic, neurogenic and memory-enhancing effects [2]. In vitro, recombinant sAPPα completely protected neurons against Aβ-dependent toxicity of dendritic spines and from the phospho-tau-dependent toxicity [5]. while human sAPPα (hsAPPα) interacts with and inhibits BACE1 (β-amyloid cleaving enzyme), resulting in a suppression of amyloidogenic APP processing and Aβ generation [6]. Meanwhile, sAPPα also shows potential for the treatment of AD in vivo. Genetic overexpression of hsAPPα suppressed β-amyloid plaques in the cortex and hippocampus in an AD mouse model [6]. Moreover, adeno-associated virus serotype 9 (AAV9)-mediated rodent sAPPα expression by intrahippocampal injection efficiently rescued deficits in spine density, synaptic plasticity and spatial memory of mice lacking both APP and amyloid precursor-like protein 2 (APLP2) [7], and in the APP/PS1 mouse model of AD [8]. The latter study also reported a modest reduction in amyloid accumulation and plaque deposition in the AD mice. Similarly, we have shown that intrahippocampal injection of a lentivirus packaging the hsAPPα construct rescued impaired hippocampal LTP and spatial memory in APP/PS1 mice, albeit without any effect on plaque load [9]. In a subsequent study, we found that intrahippocampal injection of AAV9-sAPPα rescued neurogenesis and reduced the percentage of the amyloid plaque-covered area by 24% in the dentate gyrus (DG) and by 20% in the cortex in the Tg mice [10].

In treating diseases within the central nervous system (CNS), AAVs have been applied in numerous experimental and clinical trials and have achieved some success [11]. While direct intraparenchymal injections are ideal for the treatment of some CNS diseases, this route is invasive and is less effective for diffuse encephalopathy like AD. Systemic injection of therapeutic AAV that can non-invasively exert a global CNS effect would be a promising treatment approach for AAV-based CNS disease treatment. However, the challenging aspect of viral vector treatment of neurodegenerative diseases with peripheral virus administration lies in facilitating the transportation of the therapeutic agents across the blood-brain barrier (BBB) while minimising immunogenicity and systemic toxicity, including inflammatory responses, hepatotoxicity, and potential neurotoxicity. AAV-PHP.B, the first AAV9-derived variant generated via the CREATE system, demonstrated enhanced CNS transduction and reduced peripheral tropism after systemic delivery in mice, while its successor AAV-PHP.eB achieved up to 50-fold higher neuronal transduction across multiple brain regions at low doses due to improved blood–brain barrier penetration [12].

Taking advantage of the AAV-PHP.eB [13], we found here that the peripheral administration of AAV-PHP.eB encoding hsAPPα rescued the long-term potentiation (LTP) impairment in APP/PS1 mice while also producing a profound reduction in plaque load development. Thus, AAV-PHP.eB demonstrated efficacy in crossing the BBB, transducing neural cells in widespread regions of the brain, and elevating hsAPPα levels in the tissue while exerting strong therapeutic and disease-modifying properties.

## Materials and methods

### Animals

Littermates of female C57BL/6 wild-type (WT) and APPswe/PS1dE9 transgenic (Tg) mice (The Jackson Laboratory, Bar Harbor, USA) were maintained as a colony at the University of Otago, Hercus Taieri Research Unit. Animals were group-housed in individually ventilated cages until vector delivery by tail-vein injection at 6 months of age, after which they were single-housed until euthanised at 9–10 months of age. Food and water were available ad libitum, and the cage contained one red plastic tube and shredded wood bedding as per standard housing. Animals were kept on a 12-h light: dark cycle (7 am–7 pm), and the room temperature was controlled via a thermostat set at 21 °C. All animal use was compliant with New Zealand’s Animal Welfare Act 1991 and all procedures were approved by the University of Otago Animal Ethics Committee (approval code #19/90).

Animals were randomly assigned at the time of the tail vein injection into designated groups based on treatment and genotype. All experiments and analyses were carried out in a blinded manner. After virus injection, mice were weighed every week and assessed for any physical or behavioural abnormalities for the duration of the experiment.

### Adeno-associated viral vectors (AAV)

Approval for the generation and use of adeno-associated viral (AAV) vectors was obtained from the Environmental Protection Agency (EPA), New Zealand (GMD101648). The AAV-PHP.eB-CAG-tdTomato vector was produced by Addgene (http://addgene.org/59462).

To generate the hsAPPα plasmid, the human sAPPα (NM_201414.2) coding sequence was synthesised with 2x HA 5’ tags between the signal sequence and mature N-terminus and BamHI and HindIII sites at 5’ and 3’ ends, respectively (GeneArt, Invitrogen). This construct was then digested with BamHI and HindIII and cloned into the AAV backbone of the AAV-PHP.eB-CAG-tdTomato (#59462). The AAV-PHP.eB-CAG-HA-HA-hsAPPα vector was packaged by the Brain Research New Zealand–Rangahau Roro Aotearoa Mārama platform (University of Otago, New Zealand, EPA approval GMD101648).

The vector was formulated in phosphate-buffered saline (PBS; Life Technologies, NZ) and the titer was determined by qPCR. CAG is a ubiquitous promoter and it was of identical sequence across the two vectors in the present study [14].

### Vector administration and experimental design

Mice received lateral tail vein injections consisting of a total volume of 100 µL of viral solution, containing 1 × 10¹¹ viral genomes (vg) per mouse for each virus administered. Stock viral preparations were mixed and diluted with sterile saline to reach the final injection volume.

A cohort of WT mice was injected with 1 × 10¹¹ vg/mouse of AAV-PHP.eB-tdTomato control virus in a total volume of 100 µL (*n* = 11, WT+Control). A second WT group received 1 × 10¹¹ vg/mouse of AAV-PHP.eB-CAG-HA-HA-hsAPPα together with 1 ×  10¹¹ vg/mouse of AAV-PHP.eB-tdTomato in the same total volume (*n *= 6, WT+hsAPPα). A third group involved Tg mice injected with 1 × 10¹¹ vg/mouse of AAV-PHP.eB-tdTomato in the same total volume (*n* = 10, Tg+Control). The fourth group involved Tg mice that received 1 × 10¹¹ vg/mouse of AAV-PHP.eB-CAG-HA-HA-hsAPPα along with 1 × 10¹¹ vg/mouse of AAV-PHP.eB-tdTomato in the same total volume (*n* = 7, Tg+hsAPPα). Sample sizes were based on similar previous studies in our lab [9, 10]. but with some constraints as imposed during the work conducted during the COVID-19 pandemic. Animals were quasi-randomly assigned to the groups.

Mice were anesthetized with isoflurane gas (2%, 200 mL/min). After losing consciousness, the mice were moved to the injection device, where they had constant isoflurane gas for inhalation anesthesia while on a heating pad throughout the procedure.

### Post-mortem tissue preparation

Between 3 and 4 months post-injection, mice were deeply anaesthetized with pentobarbital (150 mg/kg, i.p.) and transcardially perfused with an oxygenated ice-cold sucrose cutting solution (in mM: 210 sucrose, 26 NaHCO_3_, 20 D-glucose, 2.5 KCl, 1.25 NaH_2_PO_4_, 0.5 CaCl_2_, 3 MgCl_2_, saturated with 95% O_2_-5% CO_2_). The mice were then decapitated, the brain extracted and the hemispheres separated. One hemisphere was kept for post-mortem tissue analysis, with half of those hemispheres used for ELISAs and the remainder post-fixed in paraformaldehyde for immunofluorescence. The other hemisphere was used for electrophysiology. The assigned hemisphere for each analysis alternated between left and right for successive mice. The experimenters conducting the post-mortem analyses were blind to the experimental condition for each mouse. Note that the animals’ ages ranged from 9 to 10 months because the animals for each cohort studied were killed one by one for electrophysiology; for convenience, the animals’ age is stated as 9 months.

### Extracellular electrophysiology

The hemisphere for electrophysiological analysis was further processed by removing the cerebellum and the anterior third of the cerebral hemisphere and 400 µm horizontal slices were cut at a level through the middle of the hippocampus using a Leica VT1000 vibratome. Slices were transferred to cell culture membranes (Millipore) held in humidified incubation chambers containing artificial cerebrospinal fluid (aCSF; in mM: 124 NaCl, 3.2 KCl, 1.25 NaH_2_PO_4_, 26 NaHCO_3_, 2.5 CaCl_2_, 1.3 MgCl_2_, 10 D-glucose), which was incubated at 32 °C in a water bath continuously superfused with carbogen gas at 2 ml/min. After 30 min, the incubation chamber was transferred to room temperature and maintained for at least another 90 min prior to transfer to the recording chamber, where they were submerged in aCSF at 32.5 °C. Slices that did not display evoked field potentials were excluded from the study.

Evoked field potentials were recorded in the stratum radiatum of hippocampal CA1 as previously described [9]. Stimulation was delivered to the Schaffer collateral/commissural afferent fibres in stratum radiatum via 50 μm Teflon-insulated tungsten monopolar electrodes coupled to constant current custom-made programmable stimulators at 0.033 Hz (diphasic pulses, 0.1 ms half-wave duration) as controlled by a custom-designed Labview-based program. Recording micropipettes were filled with aCSF with resistances ranging from 2.5 to 3.5 M Ω and evoked potentials were amplified at ×500 gain with half-amplitude filter cut-off frequencies set at 0.3 Hz and 3 Hz and stored for later analysis using custom Labview software. Responses were measured as the initial slope of the field excitatory postsynaptic potential (fEPSP).

The input-output curves (I/O curve) were obtained by stimulating the slice at varying intensities (5, 10, 25, 50, 100 and 200 µA). The slices were stimulated three times at 30 s intervals at each input strength and averaged to obtain an output response for that intensity. Paired-pulse facilitation (PPF) was tested by giving the slice two consecutive stimulations at half-maximum fEPSP slope in rapid succession at varying inter-pulse intervals (20, 30, 50, 75, 100, 150 and 200 ms). The slices were stimulated three times at 30 s inter-pulse intervals and averaged to obtain an output response for that intensity.

For the LTP experiment, the stimulus intensity of the experimental pulse was adjusted to elicit a response 40% to 50% of the fEPSP slope elicited at a stimulation strength of 200 μA. Test pulses were delivered at 30 s intervals throughout the experiment. After recording a baseline for 30 min, two theta-burst stimuli (TBS) trains were delivered, 30 s apart. Each TBS train comprised 10 bursts at 5 Hz, each burst consisting of 5 pulses at 100 Hz at 200 ms intervals start-to-start. After the TBS, the recording continued for a further 120 min. The fEPSP slope for each response was calculated as a percentage change from the baseline, which was defined as the average of the last 20 sweeps before the TBS. For each mouse, 1-4 slices yielding stable, successful recordings were included in the analysis. When more than one slice from a mouse was studied, the data were averaged. For statistics reporting, n refers to the number of animals in a group.

### Histological processing

For immunofluorescence examination, the hemispheres were transferred to 4% paraformaldehyde in 0.1 M phosphate buffer (PB) and post-fixed overnight at 4 °C. The fixed hemisphere was then transferred to 30% sucrose in PB until it sank to the bottom of the solution. Sagittal sections were then cut at a thickness of 40 µm on a freezing microtome (HM325 NX; Thermo Scientific) and stored in cryoprotectant solution containing 30% sucrose and 30% ethylene glycol in PB at –20 °C until further use.

Starting from the first section lateral to the midline, in which the complete hippocampal structure could be clearly identified, every third brain section (~120 µm apart) was processed for primary antibody labelling. Brain sections were labelled as free-floating sections in 24-well plates. Sections were washed three times in 0.1 M PB before incubation for 1 h at room temperature in a blocking solution containing 0.1% Triton X-100 and 10% normal goat serum (v/v). Primary antibody labelling was done overnight at 4 °C. The primary antibodies used in this study were mouse HA-tag antibody (1:500, 901514, Biolegend), mouse purified anti-β-amyloid, 1-16 antibody (h-6E10) (1:500, 803001, Biolegend), Rabbit anti-Iba-1 (1:1000, PA5-27436, Thermo Fisher Scientific) and rabbit anti-glial fibrillary acidic protein (GFAP) (1:1000, GA524, Dako).

Brain sections were washed four times with 0.1 M PB, followed by incubation with the appropriate secondary antibodies diluted 1:1000 in blocking solution for 2 hours at room temperature. The secondary antibodies used (Thermo Fisher Scientific) included goat anti-mouse Alexa Fluor 647 (A21235), goat anti-rabbit Alexa Fluor 647 (A21244), goat anti-mouse Alexa Fluor 488 (A-28175) and goat anti-rat Alexa Fluor 488 (A-11006). Following incubation, the sections were washed in 0.01 M PBS and then agitated in DAPI (Invitrogen) solution for 5 min before mounting on glass slides (LabServ, Thermo Fisher Scientific) with antifade solution (lab-made). Coverslips were sealed with nail polish to prevent evaporation and oxidative damage.

Digital immunofluorescence images of the hippocampus and cortex were acquired using a Nikon C2+ confocal microscope system with a 20× objective lens (Nikon Instruments, Tokyo, Japan) on a NI-E microscope. Four excitation wavelengths, specifically 405, 488, 555 and 604 nm, were employed, with a pinhole aperture set at 1.2 AU. The range of emission wavelengths for each excitation wavelength was defined using the virtual bandpass function and was set at 410-482, 490-553, 562-624 and 647-720 nm, respectively. Images were captured using a Nikon DS Qi2 camera and NIS-Elements F 4.6 software (Nikon Instruments, Tokyo, Japan). Uniform imaging settings were employed for all the images obtained during the immunofluorescence experiments that involved labelling with either a single antibody or double-labelled antibodies for multiple mice. Additionally, imaging for all the animal sections was performed concurrently. The image quantification was conducted using ImageJ software (https://imagej.nih.gov/ij/). The local threshold values were optimised uniformly across all the mouse brain images to ensure consistency in the analysis.

Amyloid plaques were analysed using the ‘analyse particles’ function with 20 μm^2^ as the minimum size exclusion threshold to eliminate possible error due to nonspecific background fluorescence. The boundaries of the entire hippocampal formation, including the dentate gyrus, CA regions and the subiculum, were manually delineated as the region of interest. The extent of plaque coverage was calculated across the hippocampus, or all layers (I to VI) of the sensorimotor cortex overlying the hippocampus (hereafter referred to as cortex, ~1.1 mm × 1.1 mm) as a percentage of the total area. Three tissue sections per mouse were used for immunofluorescence analysis. Following the data collection, averaging was employed to obtain the data per mouse.

### Enzyme-linked immunosorbent assay (ELISA)

Brain hemispheres assigned to analyses by ELISA were immediately further dissected to yield hippocampus, cortex and prefrontal cortex. The latter was defined as that part of the cortex anterior to the middle cerebral artery, although the prefrontal cortex samples were not analysed in this study for logistical reasons. All tissue was snap frozen on dry ice and stored at –80 °C until analysed. Soluble proteins were extracted from hippocampal and cortical tissue by manual homogenisation using a pestle and microcentrifuge tube combination (30x strokes) in 100 µl of solubilisation buffer (5 mM phosphate buffer pH 7.4, 0.32 M sucrose, 0.5 mM phenylmethylsulfonyl fluoride (PMSF in ethanol), 1 mM EGTA, 1 mM EDTA and a protease inhibitor (cOmplete Ultra Mini Tablet, Roche) without detergent. Supernatant containing soluble proteins was isolated from the resulting mixture by centrifugation at 14,000 x *g* (two spins for 10 and 30 min, respectively, at 4 °C) and stored at –80 °C until assayed by ELISA. Extraction and solubilisation of Aβ for analysis were achieved using a previously published method [15]. Briefly, the insoluble protein pellet resulting from centrifugation to isolate the soluble protein fraction was dissolved by suspending in 4 °C formic acid and sonicating for at least 1 min until dissolved. The resulting solubilized proteins were separated from formic acid-insoluble proteins by centrifugation at 109,000 x *g* for 1 hour at 4 °C. The supernatant containing solubilized Aβ was treated with formic-acid neutralization buffer (1 M Tris base, 0.5 M Na_2_HPO_4_, 0.05% NaN_3_) and the resultant solution stored at –80 °C until analysis by ELISA. Total protein concentration was quantified using the bicinchoninic acid (BCA) assay (Pierce™ BCA Protein Assay Kit, Thermo Fisher Scientific) following the manufacturer’s instructions. Briefly, protein samples were mixed with the supplied working reagents and incubated at room temperature for 15 min. Readings were taken at 750 nm using a microplate reader (Bio-Rad iMark). Protein concentrations were calculated from a standard curve generated using bovine serum albumin (BSA) standards.

Aβ and hsAPPα concentrations of the brain samples were measured using three ELISA kits: human amyloid β (1–40) Assay Kit (IBL America, 27718), human amyloid β (1–42) Assay Kit (IBL America, 27719) and hsAPPα high-sensitivity ELISA (IBL America, 27734). The procedures were performed according to the kit instructions. The ELISA for hsAPPα was performed on the soluble fraction and ELISAs for human Aβ (1–42) and (1–40) were performed on both the soluble and insoluble fractions. Cortical hsAPPα levels were expressed as the log₁₀ picograms per milligram (pg/mg) of protein, while concentrations of both soluble and insoluble Aβ1-40 and Aβ1-42 were reported in pg/mg protein.

### Statistical analyses

GraphPad Prism 9.0 was used for statistical analysis. The data for the I/O curve and the PPF experiments were analysed using a mixed-model two-way ANOVA with repeated measures on one factor. Where post-hoc testing was appropriate, Tukey’s test for multiple comparisons was used.

For the LTP analysis, the baseline slope value for fEPSP was calculated by averaging the slopes of the last 20 responses (10 min) before TBS. The LTP was then calculated as the average slope value of the last 20 sweeps (10 min) after the TBS, expressed as a percentage change from the baseline value. Comparisons between two groups were made using the Student *t* test. Differences among treatment groups were statistically assessed using either a two-way or one-way ANOVA with Tukey’s post-hoc tests, comparing the mean of each group with the mean of every other group. For histological and ELISA data, all statistical analyses were performed using GraphPad Prism 9.0. Due to the small n’s for the histological and amyloid ELISA data, comparisons between the two groups were made using the nonparametric Mann–Whitney *U* test. All data are presented as mean ± SEM and the significance level was set at *p* < 0.05.

## Results

### Injection of AAV-PHP.eB vector into wild-type or transgenic mice did not affect weight gain

**Fig. 1:**
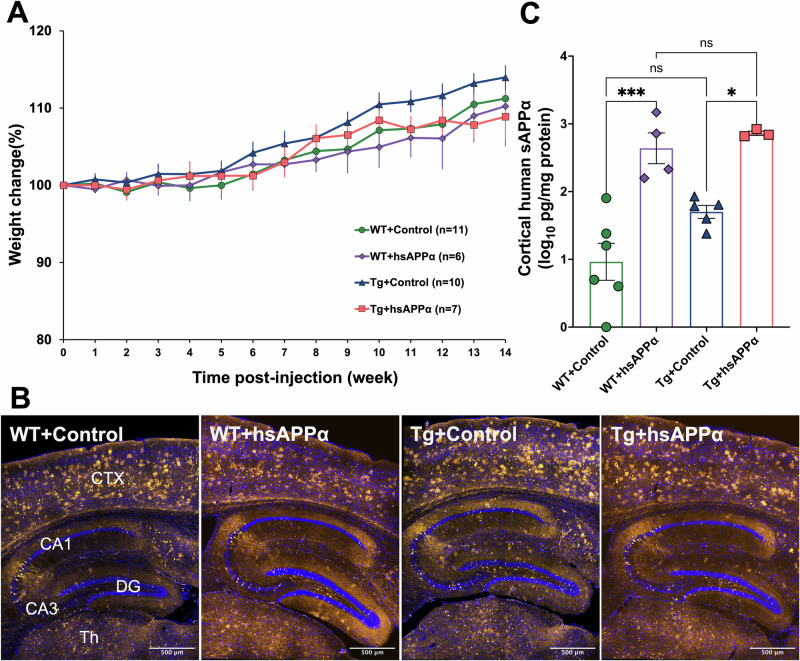
Effectiveness of brain transduction by systemic AAV injection. **A** Injection of AAV-PHP.eB vector into APPswe/PS1dE9 mice did not affect weight gain in either wild-type or transgenic mice. **B** Widespread tdTomato expression in the hippocampus, thalamus and cortex of mice injected with AAV-PHP.eB virus. Sample image from a single mouse in each group. DAPI labels nuclei (blue) and tdTomato (yellow). CTX: cerebral cortex; DG: dentate gyrus; Th: thalamus. Scale bar, 500 μm. **C** The cortical human sAPPα concentration (log_10_ pg/mg) compared across 4 groups. (WT+Control, mean ± SEM: 0.965 $\pm \,$0.274, *n* = 6; WT+hsAPPα: 2.639 $\pm \,$0.227, *n* = 4; Tg+Control: 1.700 $\pm \,$0.096, *n* = 5; Tg+hsAPPα: 2.861 $\pm \,$0.032, *n* = 3; ns, *p* >0.05, * *p* <0.05, ***p< 0.001; two-way ANOVA, Tukey’s multiple comparisons).

After the tail vein injection, mice were weighed every week (Fig. 1A). The body weight change curves showed that over time after the virus injection there was no difference between groups in their weight gain (*F*_(3, 30)_ = 0.839, *p* = 0.4832) and no time × group interaction (*F*(42, 420) = 0.762, p = 0.859), as revealed by the two-way ANOVA. Thus, all four groups of mice appeared healthy by exhibiting equivalent weight gains over the course of the experiment, as well as by the regular visual inspections of the animals and their home-cage behaviour.

### TdTomato expression in the hippocampus and cortex of mouse brain

To confirm that AAV-PHP.eB crossed the BBB successfully, fluorescence emitted by expressed tdTomato was visualised in sagittal sections of a subset of the fixed hemispheres. Extensive tdTomato expression was apparent in all tested animals from the four groups in both the hippocampus and cortex (Fig. 1B). TdTomato was expressed across the brain for the animals in each group (data not shown). The results validated previous findings that AAV-PHP.eB can cross the BBB after intravenous injection in C57BL/6 J mice [12, 13]. The results also confirmed that the tail vein injections were successful.

### Human sAPPα expression in the cortex of mouse brain

Human sAPPα concentrations were assessed by ELISA in the soluble fraction made from the cerebral cortex of the fresh frozen tissue (Fig. 1C). Due to the wide range of values obtained between control and treated groups, all data were log_10_ transformed. A two-way ANOVA revealed a significant main effect of hsAPPα virus treatment (*F*[1, 14] = 38.74, *p* <0.0001), with a significantly greater concentration of hsAPPα in the WT+hsAPPα group than in the WT+Control group (Tukey’s post-hoc test, *p* = 0.0004). Similarly, the hsAPPα concentration in the Tg+hsAPPα group was significantly greater than in the Tg+Control group (*p* = 0.02). There was no significant difference between the WT+Control group and Tg+Control group, nor between the WT+hsAPPα group and Tg+hsAPPα group. The results confirmed further that the tail vein injections were successful and that after the AAV-PHP.eB crossed the BBB, extensive cellular transduction occurred, resulting in significantly higher hsAPPα expression in the brains of the hsAPPα treatment groups compared to the control groups.

### Basal synaptic transmission

**Fig. 2:**
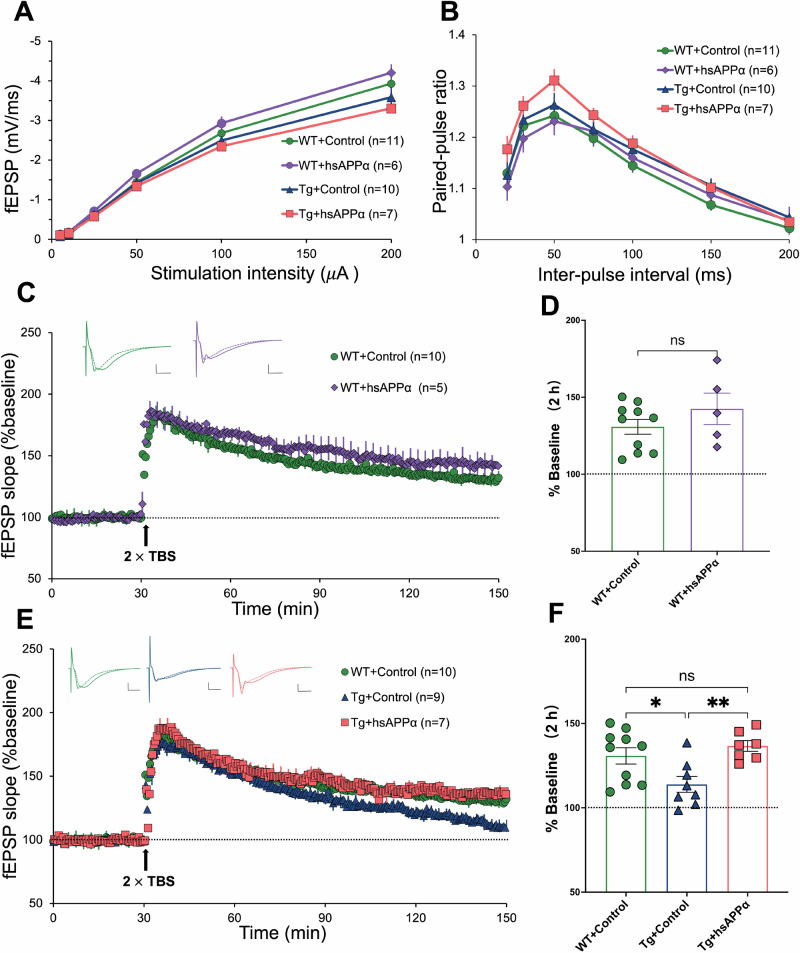
Expression of hsAPPα completely restored hippocampal CA1 LTP in transgenic mice. **A** I/O curves showing no treatment effect between groups across the stimulus intensities. **B** Paired-pulse stimulation resulted in facilitation of the second fEPSP at all inter-pulse intervals, peaking at the 50 ms interval, but there were no genotype or treatment differences in the facilitation. **C**, **E** Slices were given 2 trains of TBS and the degree of LTP followed over the next two hours. The dotted line indicates the baseline. **D** No difference in the degree of LTP at 2 h post-TBS compared between the WT+Control and WT+hsAPPα groups (WT+Control, mean ± SEM: 130.8 $\pm \,$5.1%, *n* = 10; WT+hsAPPα: 142.5 $\pm \,$10.2%, *n* = 5; ns *p* >0.05; Student *t* test). **F** The impairment in LTP persistence seen in the Tg+Control group was completely prevented by the hsAPPα treatment (WT+Control: 130.8 $\pm \,$5.1%, *n* = 10; Tg+Control: 111.8 $\pm \,$4.6%, *n* = 9; Tg+hsAPPα: 136.6 $\pm \,$3.2%, *n* = 7; ns *p* >0.05, * *p* <0.05, ** *p* <0.01; Tukey’s multiple comparisons). *Inset:* Representative waveforms are an average of 20 synaptic responses before LTP induction (dashed line) and 20 synaptic responses at the conclusion of the experiment (continuous line). Calibration bars: 1 mV, 5 ms.

The fEPSP input-output analysis for the Schaffer collateral input to area CA1 showed that increasing the input stimulation intensity generated increasing fEPSP slopes for all groups. (*F*_(1.188, 35.63)_ = 481.3, *p* <0.0001). However, there was no difference between groups (*F*_(3, 30_ = 0.953, *p* =0.427) and no group × stimulation intensity interaction (*F*_(12, 120)_ = 1.241, *p* =0.264, Fig. 2A). Thus neither genotype nor hsAPPα expression affected basal synaptic transmission in stratum radiatum of CA1. These results support previous findings that basal synaptic transmission is comparable between WT mice and APPswe/PS1dE9 mice with control vector intrahippocampal injections [8, 9]. as well as between the APPswe/PS1dE9 mice with control vector and mice with sAPPα vector treatment by intrahippocampal injection [8, 9].

Analysis of the PPF data showed the expected overall effect of the inter-pulse interval (*F*_*(2.731, 81.93)*_ = 175.4, *p* <0.0001), but no group main effect (*F*_*(3, 30)*_ = 1.632, *p* =0.203) and no group × inter-pulse interval interaction (*F*_*(18, 180)*_ = 1.279, *p* =0.206, Fig. 2B). Thus, Tg mice, with or without treatment, showed no alteration in this presynaptic form of short-term synaptic plasticity, which plays an important role in short-term adaptation to sensory inputs, transient changes in behavioural states and short-lasting forms of memory [16]. The lack of effect is consistent with the lack of PPF effects between the APPswe/PS1dE9 Tg mice and their WT littermates in previous studies [9, 17]. and with the lack of genotype and sAPPα treatment effects on basal synaptic transmission.

### Long-term potentiation (LTP)

To test the effect of hsAPPα over-expression on LTP under control conditions and to determine whether adding the hsAPPα virus in with the GFP virus caused any signs of toxicity on long-term synaptic plasticity due to the extra viral load, the levels of LTP induction from WT+Control and WT+hsAPPα groups were compared. After 2 $\times \,$TBS, both groups showed a large initial potentiation that slowly decreased without returning to baseline before the end of the two hours (Fig. 2C). A Student t-test conducted at the 2 h time point revealed no significant difference between the two groups in the degree of LTP (*p* =0.255, Fig. 2D). The lack of effect is consistent with a previous report in which recombinant sAPPα failed to upregulate a strongly induced LTP in the dentate gyrus [18]. Importantly, this finding provides further safety evidence that neither the overexpression of hsAPPα in WT mice nor the extra viral load led to adverse effects on brain functionality.

### Impact of peripherally administered gene therapy on LTP

The degrees of LTP induction from three groups (WT+Control, Tg+Control and Tg+hsAPPα) were then compared to understand the ability of hsAPPα gene therapy to rescue LTP in the Tg mice, as previously investigated in our study using intrahippocampal virus injections [9]. After the 2 $\times \,$TBS, all groups showed a large initial potentiation that steadily decreased without returning to baseline before the end of the two hours (Fig. 2E). However, the final level of LTP in the Tg+Control mice was lower compared to the WT+Control group, indicative of an impaired LTP in the control Tg mice (*F*_(2, 22)_ = 6.355, *p* = 0.0066; Tukey’s *p* = 0.032), consistent with previous research [9, 19, 20]. Importantly, there was a significant rescue of LTP by hsAPPα treatment in the Tg+hsAPPα group (*p* = 0.008 compared to the Tg+Control group), such that there was no significant difference between the WT+Control group and the Tg+hsAPPα group (*p* = 0.640, Fig. 2F).

### Amyloid plaque reduction in the hippocampus or in the cortex of Tg mice by gene therapy

**Fig. 3:**
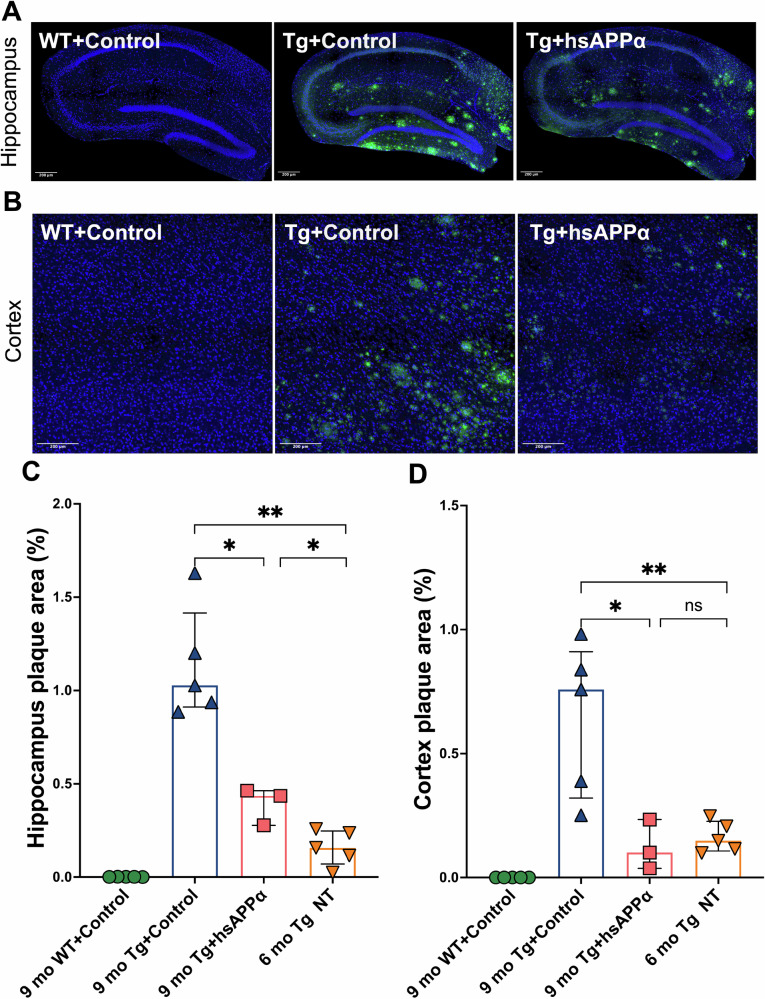
Effect of hsAPPα expression on the development of amyloid pathology. **A** Representative images of amyloid plaques in the hippocampus. **B** Representative amyloid plaque images in the overlying cerebral cortex. **C** There was a substantial development of amyloid plaque coverage in the hippocampus (% area) from 6 to 9 months of age that was significantly reduced by hsAPPα treatment (Median (IQR): 9 mo WT+Control: 0.001333 (0.000833), *n* = 5; 9 mo Tg+Control: 1.027 (0.264), *n* = 5; 9 mo Tg+hsAPPα: 0.435 (0.093), *n* = 3; 6 mo Tg NT: 0.156 (0.121), *n* = 5). **D** A similar effect was observed in the cerebral cortex (Median (IQR): 9 mo WT+Control: 0(0), *n* = 5; 9 mo Tg+Control: 0.759 (0.450), *n* = 5; 9 mo Tg+hsAPPα: 0.101 (0.098), *n* = 3; 6 mo Tg NT: 0.148 (0.091), *n* = 5). ns *p* > 0.05, * *p* <0.05, ** *p* <0.01; Mann–Whitney tests.

To understand possible mechanisms contributing to LTP rescue, a 6E10 antibody was used to label the amyloid plaques in the hippocampus (Fig. 3A) and overlying cerebral cortex (Fig. 3B). There were no plaques observed in the hippocampus of the WT+Control group. In the Tg+Control group, the hippocampus exhibited a substantial amyloid plaque load. Notably, there was a significant 65.5% reduction in the area covered by amyloid plaques in the hippocampus in the Tg+hsAPPα group (*p* = 0.036) compared to Tg+Controls. Similar results were found in the cortex overlying the hippocampus. There were no plaques observed in the cortex of the WT+Control group. In the Tg+Control group, the cortex manifested a prominent presence of amyloid plaques, with a substantial decrease in the amyloid plaque burden of 80.7% in the Tg+hsAPPα group (*p* = 0.036).

To clarify whether the effect of expressing hsAPPα was one of slowing or preventing amyloid plaque formation in the Tg mice, the amyloid plaque load was checked in an additional group of untreated Tg mice killed at the 6-month time-point (6 mo Tg NT, *n* = 5), i.e., at the time when the other groups received the viral vector injections. There was a significant increase from 6 to 9 months in the Tg mice with control treatment, both in the hippocampus (*p* = 0.0079) and in the cortex (*p* = 0.0079), indicating a substantial growth in plaque load over this period. Interestingly, following three months of peripheral gene therapy, no significant differences were observed in the cortex between the 6 mo Tg NT group and the 9-month Tg+hsAPPα group (*p* = 0.5714), but a small significant increase did occur in the hippocampus (*p* = 0.036). This result indicated that the peripheral AAV-hsAPPα gene therapy significantly slowed amyloid plaque formation.

### Hippocampal Aβ1-40 and Aβ1-42 levels are unaffected by hsAPPα overexpression

**Fig. 4:**
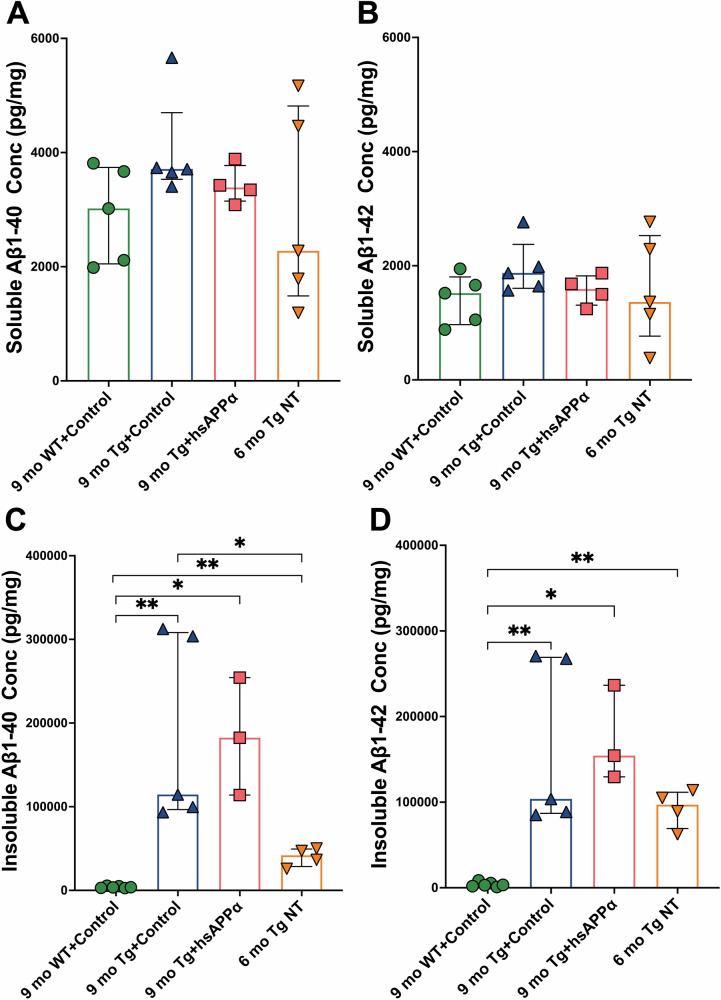
Concentrations of soluble and insoluble Aβ1-40 and Aβ1-42 in the hippocampus. **A**, **B** Neither Aβ1–40 nor soluble Aβ1–42 levels increased from 6 to 9 months of age and were not affected by hsAPPα treatment (*p* > 0.05, Mann–Whitney tests). Median (IQR): A: 9 mo WT+Control: 3019 (1556), *n* = 5; 9 mo Tg+Control: 3711 [78], *n* = 5; 9 mo Tg+hsAPPα: 3387 (260), *n* = 4; 6 mo Tg NT: 2277 (2676), *n* = 5. B: 9 mo WT+Control: 1519 (608), *n* = 5; 9 mo Tg+Control: 1874 (343), *n* = 5; 9 mo Tg+hsAPPα: 1591 (293), *n* = 4; 6 mo Tg NT: 1365 (1137), *n* = 5. **C** Insoluble Aβ1–40 levels showed a significant increase in 9 mo Tg+Control compared to 6 mo Tg NT mice, but this was not affected by hsAPPα treatment. Median (IQR): 9 mo WT+Control: 3662 (1618), *n* = 6; 9 mo Tg+Control: 114533 (204092), *n* = 5; 9 mo Tg+hsAPPα: 182480 (70218), *n* = 3; 6 mo Tg NT: 41861 (13987), *n* = 4. **D** Similar effects were seen for insoluble Aβ1–42 levels. Median (IQR): 9 mo WT+Control: 3196 (2694), *n* = 6; 9 mo Tg+Control: 103749 (178956), *n* = 5; 9 mo Tg+hsAPPα: 154343 (53487), *n* = 3; 6 mo Tg NT: 97024 (24572), *n* = 4. * *p* <0.05, ** *p* <0.01; Mann–Whitney tests.

Human amyloid-β load was also assessed by ELISA for both the soluble and insoluble fractions prepared from the hippocampus. Mann–Whitney tests did not reveal any significant differences in either soluble Aβ1–40 (Fig. 4A) or soluble Aβ1–42 (Fig. 4B) in the hippocampus.

In the insoluble fraction (Fig. 4C), there was a greater Aβ1–40 concentration in the Tg+Control group compared to the WT+Control group (*p* = 0.0043) and compared to the 6 mo Tg NT group (*p* = 0.0159). However, surprisingly, there was no significant effect of sAPPα expression on this elevated level. Note that the Aβ1–40 concentration in the 6 mo Tg NT group was less than for the Tg+Control group, but greater than for the WT+Control group (*p* = 0.0095), indicating again a developing amyloid load already by 6 months. A similar pattern of results was seen for insoluble Aβ1–42 fraction (Fig. 4D). The Tg+Control group showed an increased insoluble Aβ1–42 concentration compared to WT+Controls (*p* = 0.0043), but no significant effect of sAPPα expression on this elevated level compared to the Tg+Control group.

### Neuroinflammation analyses

**Fig. 5:**
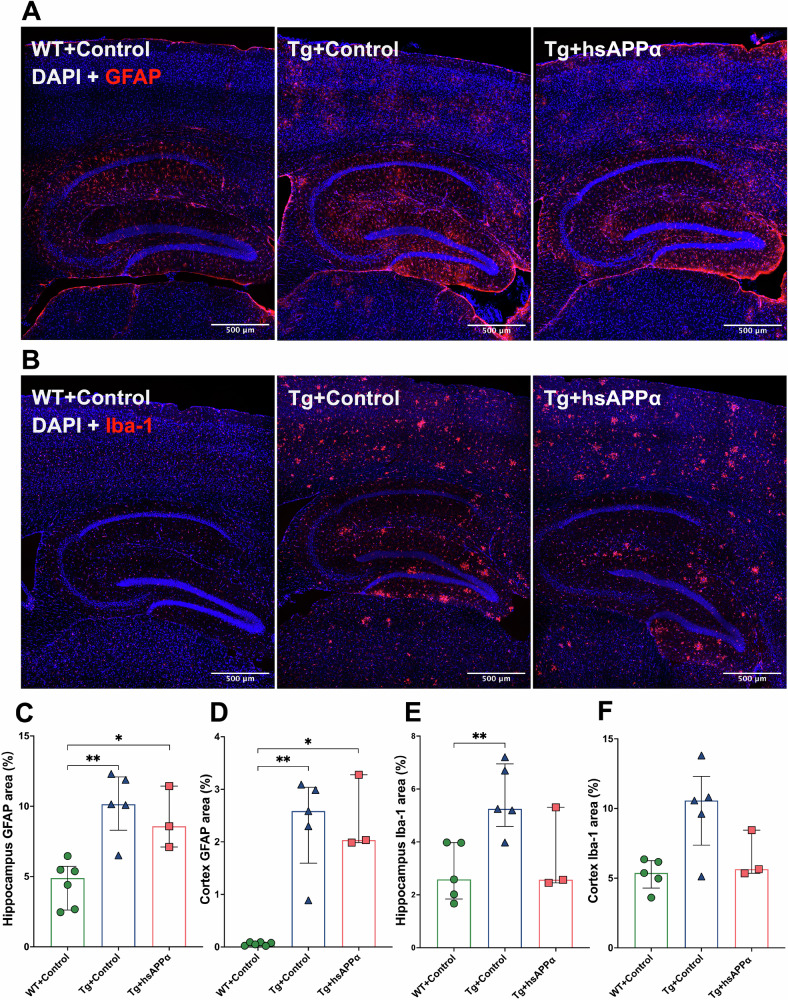
Effect of sAPPα expression on markers of neuroinflammation. **A** Representative GFAP expression in the hippocampus and overlying cerebral cortex. **B** Representative Iba-1 expression is covered by GFAP labelling in the hippocampus and cortex. There was a significant increase in the spread of GFAP labelling in Tg mice compared to WT mice for both regions of interest, but this was not affected by hsAPPα treatment. Median (IQR): **C** WT+Control: 4.89 (2.35), *n* = 6; Tg+Control: 10.14 (1.79), *n* = 5; Tg+hsAPPα: 8.57 (2.17), *n* = 3. **D** WT+Control: 0.064 (0.058), *n* = 6; Tg+Control: 2.58 (0.67), *n* = 5; Tg+hsAPPα: 2.03 (0.65), *n* = 3. **E**, **F** Bar graphs showing the percentage area covered by Iba-1 labelling in the hippocampus and cortex. There was an increase in Iba-1 labelling in Tg mice compared to WT mice in both regions of interest, albeit not significantly in the cortex. The hsAPPα treatment prevented this increase, in that the Tg+hsAPPα group was not significantly different from the WT+control group. Median (IQR): E: WT+Control: 2.58 (1.95), *n* = 5; Tg+Control: 5.25 (1.50), *n* = 5; Tg+hsAPPα: 2.57 (1.43), *n* = 3. F: WT+Control: 5.37 (1.20), *n* = 5; Tg+Control: 10.57 (1.20), *n* = 5; Tg+hsAPPα: 5.64 (1.55), *n* = 3. * *p* <0.05, ** *p* <0.01; Mann–Whitney tests.

In the hippocampus (Fig. 5A), GFAP labelling was uniformly distributed throughout the surveyed region in the WT+Control group, while a significantly higher GFAP coverage area was evident in the Tg+Control group, but no obvious alteration in GFAP coverage area in the Tg+hsAPPα group compared to the Tg+Control group. Mann–Whitney tests (Fig. 5C) confirmed that there was a significant difference between the WT+Control group and the Tg+Control group (*p* = 0.004), but no significant difference in GFAP expression between the Tg+Control group and the Tg+hsAPPα group (*p* = 0.571).

In the cortex (Fig. 5A), there was much less expression of GFAP in the WT+Control group compared to that in the hippocampus, which supported a previous finding that astrocyte GFAP expression showed regional and layer-specific differences in the rodent CNS [21]. In the Tg+Control group, an obviously increased GFAP coverage area was evident in the cortex, but again, no noticeable change in GFAP distribution in the Tg mice following treatment with AAV-hsAPPα compared to the Tg+Control group. Mann–Whitney tests (Fig. 5D) confirmed these observations, revealing a significant difference only between WT mice and Tg mice with control treatment (*p* = 0.004) and no significant difference between the Tg+Control group and the Tg+hsAPPα group (*p* > 0.999).

In the hippocampus and the cortex, Iba-1 labelling was distributed evenly throughout for the WT+Control group (Fig. 5B). The Iba-1 labelled area was noticeably higher in the hippocampus and cortex in the Tg+Control group. In particular, Iba-1 labelling exhibited extensive clustering around all the amyloid plaques in both brain regions. Consistent with the plaque load data, a significant increase in Iba-1-labelled area was observed in the hippocampus of Tg mice compared to WT mice under control treatment (*p* = 0.008; Fig. 5E). In the cortex (Fig. 5F), although an apparent increase in Iba-1 staining was evident in Tg mice, the difference between the WT+Control and Tg+Control groups was not statistically significant (*p* = 0.056). Although the Tg+hsAPPα group showed a visible reduction in Iba-1-positive area in both the hippocampus and cortex to near the level seen for the Tg+Control group, these differences were not statistically significant (*p* = 0.25 for both). On the other hand, no significant differences were also observed between the WT+Control group and the Tg+hsAPPα group in either brain region (hippocampus: *p* = 0.7857; cortex: *p* = 0.5714), highlighting that hsAPPα treatment showed a trend toward normalising microglial activation to levels comparable with WT controls.

## Discussion

Our investigation has demonstrated that expression of human sAPPα generated by a peripherally administered viral vector at an early time-point in the disease phenotype, substantially mitigated the emergence of synaptic deficits and plaque load in the brains of APP/PS1 mice modelling AD. These results were associated with the presence of elevated levels of human sAPPα using a highly specific ELISA assay. Specifically, we observed a dramatic reduction in amyloid plaque accumulation in parallel with a complete prevention of LTP deficits measured 3-4 months post-transduction of AAV-PHP. eB carrying hsAPPα. Given that the virus was administered peripherally and facilitated by the AAV-PHP.eB capsid in transversing the BBB, hsAPPα was expressed in the entire cerebrum. Taken together, these findings suggest that sustained elevation of hsAPPα concentration by a single systemic injection, initiated early during AD, may prove efficacious in at least delaying the subsequent manifestation of amyloid plaque accumulation and synaptic deficits that are typically associated with AD.

### Peripheral administration of sAPPα and the reduction of amyloid plaque in an AD mouse model

In AD, the substantial accumulation of Aβ plaques in the brain is closely associated with a cascade of pathological events—including neuroinflammation, synaptic dysfunction, mitochondrial and bioenergetic impairments and vascular abnormalities—that collectively contribute to neuronal degeneration and death [22]. Therefore, a reduction of Aβ levels in individuals with AD has been a prominent target area for the advancement of novel therapeutic agents. Lecanemab and donanemab, monoclonal antibodies targeting Aβ, were approved for clinical use in 2022 and 2023, respectively, based on their demonstrated ability to slow cognitive and functional decline in AD and their associated reductions in disease-related biomarkers [23]. These findings support Aβ clearance as a viable disease-modifying therapeutic approach for AD.

In this study, there was an obvious treatment effect on plaque load both in the cortex and hippocampus in Tg mice as compared to the control virus treatment. This pattern of results is consistent with previous treatment studies using AAV9-mouse-sAPPα with intrahippocampal injections [8, 10]. It is not surprising to find that after the intrahippocampal injection, the small AAV-HA-HA-sAPPα particles (20 nm) could diffuse efficiently throughout the hippocampus as well as from the needle tract to the overlying cortex [10]. The intrahippocampal injection of AAV9-sAPPα (rodent), however, reduced by only 23% the plaque-stained area in the hippocampus of the Tg mice [8]. While here, the plaque-stained area in the hippocampus was reduced by around 65% via systemic intravenous AAV-PHP.eB-hsAPPα administration in the Tg mice. The different homologs of sAPPα might play some role in this different level of effect, while at the same time, the promoter used could be influential. The AAV9-sAPPα (rodent) studies used the synapsin 1 promoter that restricts the transgene expression exclusively to neurons [24]. In contrast, the AAV-PHP.eB-hsAPPα virus in the current study used the CAG promoter, which could transduce both glial and neuronal cells for long-term expression [25], even though most transduction was in neurons, compared to non-neuronal cells [13]. This means that, given the ability of sAPPα to be secreted, the transduction of both glial and neuronal cells could enhance the release of extracellular sAPPα, potentially resulting in a higher level of extracellular sAPPα and thus a more efficient therapeutic effect. Alternatively, there could be a sex difference in the degree of effect, given that females were used in the present study, in contrast to males used in the above-cited ones.

It is notable that there was no treatment effect on plaque load using lentivirus-mediated delivery of sAPPα by intrahippocampal injection [9]. These differing results might be due to the different sizes and neuron tropisms of the different virus types. The larger particle size of lentivirus (100 nm) limits spread through the extracellular space, compared to the much smaller size of the AAV9 [26]. AAV9 and AAV-PHP.eB have strong neuronal tropism after intracerebroventricular injection [13], while the LV was shown to be less appropriate for infecting neurons in the cynomolgus monkey brain as compared with AAV [27].

In contrast to the results from antibody labelling of plaques, the expression of hsAPPα did not substantially affect levels of soluble or insoluble amyloid as measured in tissue samples by ELISA. This apparent discrepancy may be attributed to the sensitivity of the ELISA assay, which can detect smaller insoluble amyloid aggregates that may fall below the threshold of detection in histological analyses. It is well-established that immunohistochemical techniques are limited by their ability to detect only larger accumulations of aggregated amyloid [28]. Furthermore, while previous assays detected both human and murine amyloid-β [8]. Our study and earlier investigations [9] employed ELISAs specific to human amyloid-β. It is therefore possible that the observed effects of sAPPα treatment were predominantly on murine amyloid species, with limited or no impact on human amyloid accumulation. Finally, it is not possible with these techniques to separate parenchymal amyloid-β from that which may already be engulfed in microglia. Thus, concentrations of human and murine amyloid-β monomers, oligomers and fibrils in tissue still need to be clarified.

### Peripheral administration of sAPPα and the restoration of LTP in an AD mouse model

Synapse dysfunction and loss are key features of AD [29]. AD patients exhibit a marked reduction in synaptic density within the association cortex [30], and various Tg mouse models show impairments in LTP in acute hippocampal slices [9, 31, 32]. In this study, the APPswe/PS1dE9 Tg mice displayed the expected phenotype with a significant impairment of LTP in the Schaffer collateral pathway in hippocampal area CA1 of Tg mice at 9 months of age. Importantly, three months after the systemic injection, AAV-PHP.eB-mediated expression of hsAPPα completely prevented the LTP impairment in Tg mice. This rescue of LTP could be due in part to the reduction in plaque load and associated reduced microglial activation, as evidenced by Iba-1 labelling, which would be consistent with the role of the elevated cytokine tumour necrosis factor-α levels in mediating LTP impairment in these transgenic mice [33].

The effectiveness of sAPPα treatment in rescuing LTP in this study supports previous studies of acute or long-term sAPPα delivery in rescuing LTP in Tg mice. For example, sAPPα knock-in achieved a complete prevention of LTP impairment in APP knock-out animals [34]. Further, intrahippocampal virus-mediated overexpression of sAPPα in APPswe/PS1dE9 mice led to the successful restoration of LTP to the level observed in WT mice [8, 9], while acute administration of sAPPα in hippocampal slices also rescued LTP in Tg models [9, 20]. Interestingly, our study found that the E-LTP was not notably different between groups. Rather, it was the L-LTP that was restored to the WT level in the Tg mice groups receiving sAPPα gene therapy. Therefore, the present data are consistent with sAPPα gene therapy promoting the protein synthesis-dependent late phase of LTP [19, 35]. Taken together, these data provide further evidence for the beneficial therapeutic effects of sAPPα expression [2, 36].

### Advantages of peripheral gene therapy in AD

From a practical and forward-looking perspective, the route of administration plays a critical role in enhancing current standards of care and informing the development of future therapeutic strategies [37]. One of the most significant barriers to effective AD treatment is the presence of the BBB, which restricts access of many drug treatments to the central nervous CNS. While direct intraparenchymal administration into affected brain regions or intraventricular delivery into the CNS circulation can enhance drug bioavailability, these approaches are highly invasive, potentially painful and carry the risk of inducing damage to brain tissue. Another major challenge in AD therapy is achieving sustained therapeutic expression. Furthermore, the widespread nature of AD neuropathology complicates localised treatment efforts. Lumbar intrathecal administration via the CSF presents a promising, less invasive alternative [2]. In recent years, intranasal delivery has emerged as a non-invasive route that reduces systemic exposure and minimises adverse effects. This method is particularly promising when combined with nanoparticle-based formulations, which have demonstrated potential in crossing the BBB and improving targeting efficiency [38]. However, studies have shown that intranasal delivery of AAV vectors, such as AAV-PHP.eB or AAV9, results in limited distribution, with transgene expression largely confined to the olfactory bulb following a single dose [13]. Intravenous administration, then, represents another potential strategy for CNS drug delivery, offering a systemic route that can exploit BBB transport mechanisms for appropriate AAV vectors without the risks associated with direct brain injections.

### Limitations

This study has several limitations. First, male mice were not included, reducing the generalisability of the findings. However, sex-specific differences in AD pathology are well documented, with the prevalence of AD being higher in females [39–41]. Second, behavioural assessments were not performed, limiting functional interpretation of electrophysiological and histopathological improvements, although previous studies have shown that sAPPα delivery can rescue behavioral deficits in AD models [8, 9]. Third, the BBB-crossing capability of AAV-PHP.eB is strain-specific and ineffective in non-human primates, highlighting the need to evaluate alternative vectors such as AAV.CAP-B10 for translational studies [42, 43]. Additionally, while hsAPPα reduced amyloid plaque burden, it did not significantly alter astrocytic or microglial activation, warranting further investigation of glial subtypes and neuron–glia interactions with greater numbers of subjects. Finally, systemic immune responses to viral vector administration were not assessed, which may influence transduction efficiency and safety. However, no ill effects were visually evident, the mice gained weight in a normal fashion and basal synaptic functions were not affected.

## Conclusions

This work has demonstrated the feasibility of employing a viral vector for the peripheral administration of sAPPα as a therapeutic intervention for AD. This innovative approach obviated the requirement for invasive and precision-demanding surgical procedures aimed at overcoming the BBB, thereby mitigating potential associated damage. The findings of this study represent, therefore, a substantial advancement towards the achievement of a non-invasive therapeutic strategy for AD through systemic delivery.

## Data Availability

Data are available from the corresponding author upon reasonable request.
